# Percutaneous Nephrolithotomy: Current Clinical Opinions and Anesthesiologists Perspective

**DOI:** 10.1155/2016/9036872

**Published:** 2016-03-27

**Authors:** Indira Malik, Rachna Wadhwa

**Affiliations:** Department of Anesthesiology and Critical Care, Dr. Baba Saheb Ambedkar Hospital, Rohini, Delhi 85, India

## Abstract

Percutaneous nephrolithotomy (PCNL), a minimally invasive method for removal of renal calculi, was initially started in the 1950s but gained popularity about two decades later and has now become standard practice for management. There has been an immense improvement in technique and various guidelines have been established for treatment of renal stones. However, it has its own share of complications which can be attributed to surgical technique as well as anesthesia related complications. PubMed and Google search yielded more than 30 articles describing the different complications seen in this procedure, out of which 15 major articles were selected for writing this review. The aim of this review article is to describe the implications of the complications associated with PCNL related to the anesthesiologist. The anesthesiologist is as much responsible for the management of the patient perioperatively as the surgeon. Therefore, it is mandatory to be familiar with the various complications, some of which may be life threatening and he should be able to manage them efficiently. The paper also analyses the advantages and drawbacks of the available options in anesthesia, that is, general and regional, both of which are employed for PCNL.

## 1. Introduction

Percutaneous nephrolithotomy (PCNL) is an established, minimally invasive procedure for removal of renal calculi more than 2 centimetres in size. In 1976, Fernstorm and Johansson reported the removal of renal calculus through a nephrostomy tract for the first time [1]; since then PCNL has become the most common procedure performed for the management of renal stones. It facilitates a direct approach to the calculus while the kidney and surrounding structures are subjected to lesser trauma as compared to the open approach, and hence a great deal of surgical expertise is required for percutaneous access to the kidney and stone removal. Many changes and modifications have been done to minimise morbidity, analgesic requirements, and duration of hospitalization, including use of regional blocks, single step dilatation, “Mini-Perc” technique, tubeless PCNL, and sandwich therapy [2–4].

Though the entire surgical team shoulders the responsibility for the outcome, the role of the anesthesiologist is not less. They not only help in providing optimal working conditions for the surgeon, but also recognise and deal with the complications related to anesthesia and the procedure as a whole.

## 2. Method for Data Collection and Synthesis

The keywords PCNL, complications, and anesthesia were searched in PubMed and Google and 30 articles were retrieved. Out of these, 15 articles were considered pertinent to our review. The data from these articles was retrieved and results of the related studies are compared and compiled to synthesize this review.

## 3. Background

The incidence and prevalence of renal calculi have increased globally across all ages, sex, and race, probably due to change in dietary habits and global warming [5]. The conventional and perhaps the oldest method of removing renal stones was open nephrolithotomy. Later, with the advent of ureteroscopes, ureteric stones were removed with the help of dormia baskets. Percutaneous method of removing renal calculi was first described in the 1950s but it was actually performed almost two decades later in the 70s and 80s for routine removal of renal stones. Simultaneously, other procedures for removal of renal stones, such as extracorporeal shock wave lithotripsy (ESWL), also came into vogue and PCNL remained an underutilized procedure, until the last decade, which witnessed a surge in this specific procedure vis-à-vis improvement in technique, increased prevalence of renal stones, and delineation of clear cut indications for PCNL [7]. Currently, PCNL is the procedure of choice for managing kidney stones and continues to evolve and has largely replaced open stone surgery. Advances in technology and equipment have resulted in stone removal with less morbidity, shorter convalescence, and reduced cost compared with open surgery [8].

Currently, the indications for PCNL include large size renal calculi (>1.5–2 cm), staghorn calculi, upper tract calculi not responding to other modalities of treatment, lower pole stones, cystine nephrolithiasis, and stones in anatomically abnormal kidneys [7]. Generally two approaches are followed for PCNL.

*Standard PCNL*. The procedure is performed with the patient in prone position. A small incision 1.3 cm is given on the back overlying the affected kidney and a track is created from the skin to the kidney. It is then enlarged using a series of Teflon dilators or bougies with a sheath being placed over the last dilator to hold the track open. After this, nephroscope (fibre optic light source with two additional channels for renal visualisation and irrigation) is inserted; smaller stones may be removed with the help of a device with a basket at its end while larger stones may be fragmented by a Holmium laser lithotripter, ultrasonic or electrohydraulic probe. After removing the stones, a catheter is placed to drain the urinary system and a nephrostomy tube is placed to drain the fluid from the kidney to a drainage bag. They are usually removed before the patient is discharged. 

*Mini PCNL*. It is performed with miniaturized nephroscope and has been found to have 99% efficacy in removal of stones 1–2.5 cm in size. Though not useful for larger calculi, it offers the advantages of shorter operating and recovery time and fewer complications.

Like all minimally invasive procedures, PCNL too has its share of complications, related to both the procedure and the underlying pathology. Early recognition and management of many of these complications reduce the morbidity and mortality considerably. A multicentre study of 5803 patients, conducted by the Clinical Research of the Endourological Society (CROES), reported an overall complication rate of 21.5%, using the modified Clavien system [9], which was developed in 1992 and later modified in 2004, for classification of complications [10, 11] and comparison of complication rates in radical prostatectomy and cystectomy. The modified Clavien system of classification of complications was first adopted by Tefekeli et al. in 2008 to stratify complications following PCNL [12] (Table 1).

## 4. Anesthetic Technique

There has been considerable debate regarding the ideal anesthetic technique for PCNL. The procedure is usually performed under general anesthesia (GA) and the published literature regarding the use of spinal anesthesia for PCNL is sparse [8]. Over the years, both these techniques have been used and they have their advantages and drawbacks. The advantages offered by GA include safety as the patient's airway is secured in prone position, feasibility to control tidal volume during percutaneous access puncture to minimise injury to the pleura and lungs, and prolonged anesthesia duration allowing the surgeon to make multiple and higher punctures with minimal patient discomfort, especially in cases with large stone load.

Regional anesthesia (RA) for PCNL was first described in 1988 [13]. Since then, a few studies have been done regarding use of regional anesthesia for PCNL [14–19]. It has its own merits in the form of less postoperative pain, less blood loss, and early recovery and discharge thereby reducing stay in the hospital.

A randomized, controlled trial including 200 patients compared the efficacy of GA versus RA in PCNL patients and concluded that both the groups had comparable intraoperative hemodynamics. However, postoperative Visual Analogue Score (VAS) scores were comparatively less after one hour postoperatively in the RA group while the GA group patients received analgesics within the first postoperative hour itself. Therefore, the consumption of systemic analgesics was greater in the patients who underwent GA. Incidence of postoperative nausea and vomiting was significantly higher in the GA group, but the overall patient satisfaction was also better. The patients who received RA had an increased incidence of shivering but the procedures were completed successfully without conversion to GA [20].

Mehrabi and Shirazi evaluated the intraoperative and postoperative anesthetic and surgical outcomes in patients who underwent PCNL under spinal anesthesia in prone position and concluded that spinal anesthesia is not only safe and effective for performing PCNL, but it is also a good alternative for GA in adult patients [16]. Borzouei et al. did a large study regarding the use of spinal anesthesia in PCNL and reported that spinal anesthesia is feasible, safe, and well tolerated especially in elderly patients with significant comorbidities such as pulmonary disease [15].

Other authors have previously compared the two techniques and found that while RA provided the advantage of better analgesia and shorter recovery times, GA was more comfortable for the patients in prone position and also safer, in case the procedure was prolonged [21, 22]. Kuzgunbay et al., 2009, found no significant difference between the GA and RA groups in terms of operative time, success rate, hemoglobin level, hospital stay, and complications. However, patients' satisfaction was higher in the RA group [17]. In a large retrospective study, involving 1004 patients, complications were graded according to Clavien classification and comparison of the two groups was done which revealed that the overall rate of complications was greater in the GA group [23].

The many specific anesthesia concerns in PCNL mandate a fine coordination between the surgical and anesthesia teams for optimal results. The choice of anesthesia depends, to a great extent, upon the patient's preference, the position for surgery favoured by the surgeon, surgical expertise, and estimated time of the procedure determined by the stone size, number, and location. The anesthesiologist should be fully aware of all the possible complications, irrespective of the choice of anesthetic technique, that occur intraoperatively as well as postoperatively.

### 4.1. Preoperative Considerations

Patients belonging to varying age groups may present for PCNL. Elderly patients may have many comorbidities like ischemic heart disease, respiratory dysfunction, and diabetes while young children may be highly uncomfortable and uncooperative. Hydronephrosis causing deranged renal functions and sepsis, in addition to the above, may add to the above. It is essential to communicate with the surgical team and confirm the adequacy of renal function by intravenous pyelogram (IVP), DMSA, or DTPA scan [24]. DMSA (dimercaptosuccinic acid) is a renal imaging tool to evaluate renal structure and morphology and DTPA (diethylene triamine pentaacetic acid) renal scan is performed to evaluate the blood supply and function and excretion of urine from the kidneys. This test can also find out what percentage each kidney contributes to the total kidney function. There is no indication for performing PCNL if the kidney is nonfunctional; such patients should be taken up for nephrectomy. Stabilization of the existing comorbidities in the preoperative period reduces intra- and postoperative complications, overall morbidity, and mortality. There should not be any active focus of infection preoperatively.

### 4.2. Intraoperative Considerations

Although GA has been considered to be the safest technique for PCNL worldwide, it has its own hazards like accidental extubation and kinking of endotracheal tube (ET) during positioning of the patient; hence, it is desirable to use a reinforced ET tube or an oral airway along with a regular ET tube and the tube should be firmly secured. Position of bolsters should be carefully checked to allow unhampered ventilation. Torsion of the neck veins may lead to facial edema, ocular edema, and ecchymosis.

Care should be taken to avoid pressure on the eyeballs in the prone position as this may lead to postoperative visual loss; if the external pressure on the globe exceeds the mean arterial pressure (MAP), perfusion to the optic nerve is hampered, leading to blindness. Pressure on the pinna should be avoided as it can cause pressure necrosis. In female patients, the breasts should be positioned medially to avoid pressure necrosis. The arms should be abducted and brought upwards with the elbows flexed equally to prevent overstretching of the brachial plexus on either side. All the pressure points like the elbows, wrists, knees, and ankles should be adequately padded to prevent peripheral nerve injuries.

When RA is used, many of the issues related to positioning are resolved as the patients are conscious and can position himself/herself in the prone position according to their comfort. However, there is a risk of sudden hypotension after making the patient prone. Patient discomfort increases with the duration of the procedure and the surgeon may not feel comfortable in making skin punctures, especially those close to the 11th rib, if the patient is unable to coordinate breath holding at that time.

## 5. Complications Related to Surgical Procedure and Their Anesthetic Implications

Various authors have studied complications related to PCNL per se (Table 2). Besides the definite risk of injury to the surrounding organs and major blood vessels, these patients are also at considerable risk for sepsis. Each of these complications adds to the morbidity and sometimes mortality of patients. The majority of the complications occur in the intraoperative and immediate postoperative period; therefore, the anesthesiologist must be aware of the complications and be well equipped to deal with them effectively and minimise morbidity.

The overall rate of pleural injury ranges between 0.3 and 1% during percutaneous access puncture for PCNL. It may manifest as hydrothorax, pneumothorax, or hydropneumothorax and 64% of them may require chest tube drainage [25]. Since they are mostly diagnosed in the immediate postoperative period with shortness of breath, fever, and radiological evidence of pleural injury [26], it is desirable to fluoroscopically monitor the chest during the procedure and obtain a chest radiograph in the postoperative period.

Chances of pleural and lung injury are higher during upper pole access due to the close proximity of these structures. Preminger et al. (2005) reported 16% pleural injuries with supracostal approach as opposed to 4.5% with subcostal approach [27]. Ideally, the working sheath should be inserted under the 11th rib and above the 12th rib with ventilatory standstill; however, punctures above the 11th rib increase the risk of intrathoracic complications to 23.1% as compared to 1.5–12% for punctures above the 12th rib; the risk is 0.5% for subcostal approach [28]. Pulmonary injuries are likely to occur in 29% of cases on the right side and in 14% of cases on the left side [29]. A higher nephrostomy tract predisposes the patient to greater risk of incurring intrathoracic injury; therefore, when multiple or higher punctures are required to remove a greater stone load, GA would be safer and desirable and vigilance is required throughout the procedure, for raised airway pressures and end tidal CO_2_, and falls in oxygen saturation indicating pleural or lung injury which should be managed promptly by maintaining ventilation with 100% oxygen and if pneumothorax is diagnosed intraoperatively, placement of chest tube.

Adjacent solid organs are also at risk for injury during percutaneous access approach. Hepatic or splenic injury may occur in case of coexisting hepatomegaly or splenomegaly. Currently there is no general consensus regarding management of splenic injuries; however, the majority of articles support conservative management. Similarly, liver injury is managed best with conservative approach. However, all gall bladder injuries necessitated cholecystectomy, the timing of diagnosis being the decisive factor between laparoscopic and open techniques [30].

Injuries to hollow organs like the small bowel and colon have been reported in 0.2–1% of cases [30, 31]. Bowel injury is more complicated to deal with due to intraperitoneal involvement and requires laparotomy. Gall bladder or small bowel perforation may lead to secondary peritonitis, sepsis, and septic shock with a mortality rate of 30%, if not managed early [32].

El-Nahas et al. retrospectively reviewed 5093 PCNL cases of which 15 patients (0.3%) suffered colonic perforation. Out of these 15 patients, 66.6% had left percutaneous access and 80% underwent lower calyceal puncture. Advanced age, thin built, female sex, and horse shoe kidney and cases with previous bowel or renal surgery were also independent risk factors for colonic injuries [33, 34].

In addition, the great vessels are also at risk for damage during percutaneous access puncture, in about 0.5% of cases [31]. Multiple attempts at initial percutaneous access, upper pole access, inexperienced surgeon, solitary kidney, and staghorn calculus may increase the risk of bleeding. The potential for massive bleeding during percutaneous access to the renal pelvis may occur due to the close proximity of the major hilar blood vessels and scanty parenchymal tissue to provide tamponade in case of injury [7]. Initial bleeding during the access may be from the renal capsule or parenchyma and is venous in nature and hence is controllable with balloon dilator and Kaye's tamponade catheter. Adequate hydration, large nephrostomy tube, and mannitol may also be helpful. Improved techniques have significantly reduced transfusion rates and interventional control of delayed hemorrhage is now possible [6].

A series was conducted by El-Nahas et al., 2007, in 39 patients of PCNL complicated with severe bleeding who underwent arteriography and embolization. Pseudoaneurysms and arteriovenous fistulae were the most common causes of hemorrhage in them. Superselective angioembolization successfully controlled bleeding in 92% of cases, remaining cases required exploration, and one patient underwent nephrectomy [35]. A retrospective study by Srivastava et al., including 1854 patients for PCNL, reported major bleeding requiring angioembolization in 1.6% of cases. Pseudoaneurysm was found in 48.1% of bleeding patients on renal arteriography and 91.6% were successfully treated with angioembolization; the common factor between all the patients was increased stone size (>4.1 cm) [36]. Other factors responsible for increased risk of bleeding are diabetes mellitus, utilization of mature nephrostomy tract, concomitant surgical complications, access tracts passing through atrophic parenchyma, and the modality used for guiding access (fluoroscopy versus ultrasound) [37, 38].

Injury to the renal collecting system occurs in 8% of patients, causing extravasation and absorption of irrigation fluid leading to electrolyte disturbances, altered mental status, and intravascular volume overload. Decrease in the drainage of irrigation fluid, abnormal hemodynamic parameters, and direct visualisation of perinephric fat indicate renal collecting system injury. Perforation of the renal pelvis may necessitate immediate cessation of the surgical procedure and adequate drainage through ureteric stent, nephrostomy tube, or percutaneous drain. Use of isotonic solutions for irrigation, fluoroscopic guidance, and continuous or open irrigation system can minimise this type of complication [39].

The anesthesiologist should remain vigilant and cautious to detect electrolyte imbalance and features of volume overload, which is easier to detect in patients under RA. It may manifest as irritability and discomfort followed by altered hemodynamics and ECG changes [35]. Under GA, hemodynamic changes indicate possible volume overload and should be promptly managed by restriction of intravenous fluids and diuretics. Monitoring of serum electrolytes and blood gases should be done both intra- and postoperatively.

Renal collecting system obstruction may occur rarely as a result of ureteric avulsion or stricture, leading to nephrocutaneous fistula, hydrocalyx, and hydronephrosis [40, 41]. Prolonged operative time, extended nephrostomy tube drainage, and large stone burden are some of the predisposing factors.

Renal dysfunction following PCNL is very rare and may occur secondary to other operative complications. Intraoperative or postoperative bleeding and subsequent requirement for angioembolization may compromise renal perfusion and lead to renal parenchymal infarction. Transient rise in serum creatinine may occur in 1% of patients and those who had an uneventful intra- and postoperative course exhibit <1% renal parenchymal damage [7].

Postoperative fevers may occur transiently in 30% of patients undergoing PCNL while sepsis may develop in 3% of cases. According to Korets and colleagues, 2011, 9.8% of cases of PCNL developed severe infection preceded by clinical features of systemic inflammatory response syndrome (SIRS). Univariate analysis revealed that female patients with multiple punctures, stone burden >10 cm^2^, struvite calculi, positive pelvic urine, and stone cultures were at risk for development of SIRS while multivariate analysis identified a total stone load >10 cm^2^, positive pelvic urine or stone cultures, and multiple pelvic punctures as potential risk factors [7, 42]. Since the reports of the cultures collected intraoperatively are not immediately available in the postoperative period, it becomes difficult to guide appropriate antibiotic therapy [42]. In another study by Margel et al., 2006, 25% of patients who had positive stone cultures had negative voided urine culture preoperatively [43]. The appropriate management of post-PCNL sepsis becomes difficult in this scenario as the correct antibiotic therapy may not be followed. Around 30% of the patients who develop features of SIRS require ICU care, with morbidity and mortality being high [42].

With a high index of suspicion for sepsis, broad spectrum antibiotics should be considered within one hour of admission to ICU and if required, mechanical ventilation with lung protective strategy should be considered. Inotropic support should be initiated in case of persistent hypotension (MAP < 65 mmHg). Urine output, mixed venous saturation, blood gases, and other routine parameters should be monitored. Once the patient's condition is stabilized and sepsis is under control, gradual weaning-off from inotropes and ventilatory support should be considered.

Death following PCNL is very rare and incidence ranges between 0.1 and 0.7%, occurring secondary to complications like pulmonary embolism, myocardial infarction, and sepsis [6, 44].

## 6. Conclusion and Recommendation

Though PCNL is a routinely performed, minimal access surgery, yet it may have many devastating and life threatening complications. It is safe to conduct the procedure under GA for complicated or prolonged procedures. RA is preferred only when the surgical team has a high degree of expertise and the procedure is uncomplicated. The anesthesiologist must be familiar with the various complications and their appropriate management. Effective communication between the surgical and anesthesia teams is desirable to formulate the correct perioperative management plan for every patient.

## Figures and Tables

**Table 1:** Classification of surgical complications according to the modified Clavien grading system [6].

Grade	Description of complication
I	Any deviation from the normal postoperative course without the need for pharmacological treatment or surgical, endoscopic and radiological interventions. Allowed therapeutic regimens are as follows: drugs as antiemetics, antipyretics, analgesics, diuretics, electrolytes, and physiotherapy. This grade also includes wound infections opened at the bedside.

II	Requiring pharmacological treatment with drugs other than such allowed for grade I complications. Blood transfusion and total parenteral nutrition are also included.

III	Requiring surgical, endoscopic, or radiological intervention.

IIIa	Intervention under regional anesthesia.

IIIb	Intervention under general anesthesia.

IV	Life threatening complication requiring ICU management.

IVa	Single organ dysfunction (including dialysis).

IVb	Multiorgan dysfunction.

V	Death.

Modified and adapted from [6].

**Table 2:** Studies on complications of PCNL.

Sl number	Author	Transfusion	Massive hemorrhage	Fever	Sepsis	Colonic injury	Pleural injury	Extravasation of urine	Mortality
1	El-Nahas et al. 2012 (*n* = 241)	16%	2%	1.2%	0.4%	NA	2.4%	8%	0.4%

2	Mousavi-Bahar et al. 2011, (*n* = 671)	0.6%	1.5%	1%	0	0.3%	0.7%	5.2%	0.3%

3	De la Rossette et al. 2011, (*n* = 5803)	5.7%	NA	10.5%	NA	NA	1.8%	3.4%	0.3%

4	Shin et al. 2011, [6] (*n* = 88)	6.9%	1.4%	11%	0.6%	0.7%	1.1%	0.4%	0.4%

5	Rana et al. 2007, (*n* = 667)	1.49%	0.14%	NA	1.79%	0	0.14%	NA	0

6	Osman et al. 2005, (*n* = 315)	0	0.3%	32%	0.3%	0	0	NA	0.3%

7	Lee et al. 1987, (*n* = 582)	11.2%	NA	22.4%	0.8%	0.2%	3.1%	7.2%	0.3%
